# Supplementary material for: Attention to detail: A conditional multi-head transformer for traffic sign recognition
Source: PLoS One. 2025 Nov 4;20(11):e0335341. doi: 10.1371/journal.pone.0335341 (PMC12585093; doi:10.1371/journal.pone.0335341)
Supplement: Attachment — Submitted filename: Response to Reviewers.docx [file pone.0335341.s003.docx]

**REVIEWERS COMMENTS:**

**Reviewer#1, Concern # 1: The sentence structure in abstract need to be improve i-e. “While issues related to object recognition, such as traffic sign recognition… “ is too lengthy and contains overly technical descriptions. Abstract should instead briefly state the problem, the novelty of the approach, key results, and implications.**

**a. Phrases like “gratuitous” are misused and should be replaced with clearer academic wording.**

**Author response:** Thank you for the valuable comment.

**Author action:** The abstract has been restructured for conciseness, technical wording refined, and “gratuitous” replaced with clearer academic phrasing.

**Reviewer#1, Concern # 2: The introduction is informative but contains repetition (e.g., multiple mentions of misclassification risks and safety-critical concerns). Restructuring into a concise flow would improve readability.**

**a. Sentence like “…a misplaced label can be immensely disastrous will make this more problematic” is grammatically awkward and should be revised for clarity.**

**Author response:** Thank you for highlighting this. We agree that the introduction required refinement.

**Author action:** The introduction has been restructured to remove repetitions, improve flow, and the awkward sentence has been revised for grammatical clarity.

**Reviewer#1, Concern # 3: The listed contributions are significant; however, they should be rewritten more concisely and distinctly in a separate heading, avoiding overlap with methodology descriptions.**

**a. Also, explain the third contribution, “Proposed CViT incorporates a specialized attention module… “What is meant by specialized attention module here? And how is it specialized?**

**Author response:** Thank you for this helpful observation.

Author action: Contributions have been presented concisely under a separate heading “2 Research Contributions “, and the specialized attention module has been explained as a dynamic mechanism that adjusts attention weights across traffic sign types.

**Reviewer#1, Concern #4: The methodology is presented in detail with equations and figures, which is commendable. However:**

**a. Several equations (e.g., Eq. (1), Eq. (2)–(4)) lack variable definitions. Every parameter (e.g., P, C, d) should be explicitly explained, as this can confuse readers unfamiliar with the notation.**

**b. In Eq. (5) bg is not described, what is bg and what is its use in Eq. 5?**

**c. In 3.1.1 “Then each patch x_i for(i=1,2,…,N) are flattened into a vector of size P^2∙C, where C is the number of channels, for example, for RGB images C=3.” is mentioned but again repeated at the end of 3.1.2. It needs explanation, why is it repeated again?**

**Author response:** Thank you for the detailed suggestions. We acknowledge the need for clarity.

**Author action:** Variable definitions have been added to all equations, bg has been defined and its role explained in Eq. (5), and the repeated flattening step has been clarified to indicate its connection to positional encoding.

**Reviewer#1, Concern # 5: Several grammar issues (e.g., “Figure 6 demonstrate” should be “demonstrates”). The manuscript needs a thorough language polish.**

**Author response:** Thank you for pointing this out.

**Author action:** The entire manuscript has undergone thorough proofreading and grammar polishing.

**Reviewer#1, Concern # 6: Ensure that acronyms (e.g., GAP, FFN, MHA) are introduced at first use and used consistently throughout.**

**Author response:** Thank you for the observation.

**Author action:** Acronyms have been introduced at first mention and standardized throughout the paper.

**Reviewer#1, Concern # 7: Equation numbering is inconsistent, like in Eq. 1 to Eq. 10, parentheses “()” are used, but in Eq. 11-12, square braces “[]” are used.**

**Author response:** Thank you for noticing this.

**Author action:**  Equation numbering has been corrected and made consistent across the manuscript.

**Reviewer#1, Concern # 8: In Section 3.1.1, the phrase “as e.g., 224×224” is unpolished. Suggested edit: “e.g., resized to 224×224 pixels.”**

**Author response:** Thank you for highlighting this.

**Author action:** The phrase has been revised to “These images are first resized to a fixed resolution as $H*W$ e.g., 224×224 pixels.

**Reviewer#1, Concern # 9: Reference formatting is inconsistent: some include DOIs, others do not. Standardize per journal guidelines.**

**Author response:** Thank you for pointing this out.

**Author action:** All references have been standardized according to the journal’s guidelines, with DOIs included where available.

**Reviewer#2, Concern # 1: 1) (a) The related work section is extensive; however, the flow is occasionally interrupted by abrupt transitions. In addition, it lacks critical analysis and a discussion of the limitations of prior studies. It is also unclear why ViT is specifically preferred or emphasized.**

**(b) Furthermore, please add a new table in the related work section that summarizes the pros and cons of previous methods and clearly illustrates how your contribution relates to them and provides improvements.**

**Author response:** Thank you for this constructive feedback.

**Author action:** The related work section has been restructured with smoother transitions, critical analysis, and justification of ViT usage. A new summary table (Table 1) comparing prior methods with our contribution has been included.

**Reviewer#2, Concern # 2: In Figure 4, what is *N? is it mistaken? if not, please properly explain its working or importance in Figure 4.**

**Author response:** Thank you for the observation.

**Author action:** *N has been defined as the total number of patches, and its role in Figure 4 has been explained in the caption and text.

**Reviewer#2, Concern # 3: In 3.2.4, “The Residual & Normalization after Attention, Feed-Forward Network (FFN), and Residual & Normalization after FFN are mathematically represented …” are these the modified equations or just the basic ones? If modified, then please explain them. And if these are just the same as the basic Vision transformer, then please include references.**

**Author response:** Thank you for pointing this out.

**Author action:** We clarified that these equations are standard transformer equations and added appropriate references.

**Reviewer#2, Concern #4: The algorithm of the proposed Conditional Vision Transformer is good and well describes the whole methodology. The fail-control mechanism is a key strength, but its explanation is somewhat limited, and it is not discussed in the abstract.**

**(a) Table 7 includes valuable metrics, but abbreviations (e.g., “Critical Omissions,” “Error Reduction”) should be explained in captions.**

**(b) Figure 5 needs to be explained properly with details of metrics and parameters.**

**Author response:** Thank you for emphasizing this important point.

**Author action:** Fail-control explanation has been expanded in the methodology and included in the abstract. Table 7 caption now explains abbreviations, and Figure 5 description has been enhanced with metric and parameter details.

**Reviewer#2, Concern # 5: There are typos in the experimental section may be like “color jitter”, “WeightedRandomSampler”. It also misses the details about the training-testing ratio for the experiments.**

**(a) Fail Control Mechanism result values and parameters must be explained and discussed properly.**

**(b) In Result Section T1, T2, …. Are mentioned what are these? Mention and explain them properly. Also, in Table 1 and Table 3 why only top 5 classes or least 5 classes are mentioned. Description about other classes?**

**Author response:** Thank you for the detailed review.

**Author action:** Typos have been corrected, train-test split (80:10:10) added, Fail Control parameters explained, T1–T43 notation clarified as class IDs, and explanation provided for focusing on top/bottom 5 classes while noting full results are available.

**Reviewer#2, Concern # 6: The acknowledgment, funding, and data availability sections are included, but their formatting should match journal requirements.**

**Author response:** Thank you for pointing this out.

**Author action:** These sections have been reformatted to align with journal requirements.

**Reviewer#2, Concern # 7: Some recent and relevant works could be included to broaden the comparison and highlight novelty.**

**Author response:** Thank you for this valuable suggestion.

**Author action:**  Five recent works (2024–2025) on lightweight ViTs, adversarial robustness, autoencoder defenses, cross-domain TSR, and multimodal methods have been added to the related work section with critical discussion.

**Reviewer#2, Concern # 8: On Page 2, Line 28: the phrase “will make this more problematic” is awkwardly worded. Consider rephrasing as “further exacerbates the problem.”**

**Author response:** Thank you for the suggestion.

**Author action:** The phrase has been revised.

**Reviewer#2, Concern # 9: Some sentences are very long; breaking them into shorter sentences would enhance readability.**

**Author response:** Thank you for pointing this out.

**Author action:** Long sentences have been revised and broken into shorter, clearer statements to improve readability.
