# Supplementary material for: Attention to detail: A conditional multi-head transformer for traffic sign recognition
Source: PLoS One. 2025 Nov 4;20(11):e0335341. doi: 10.1371/journal.pone.0335341 (PMC12585093; doi:10.1371/journal.pone.0335341)
Supplement: Attachment — Submitted filename: Response_to_Reviewers_auresp_2.docx [file pone.0335341.s004.docx]

**REVIEWERS COMMENTS:**

**Reviewer#1, Concern # 1: The abstract is clear but slightly long. Consider shortening by removing repeated phrases, especially where results are restated in the last two sentences.**

**Author response:** Thank you for the valuable comment.

**Author action:** The abstract has been shortened by removing repeated phrases and redundant result restatements.

**Reviewer#1, Concern # 2: The diagrams are informative, but the resolution and font size are low. Please improve image quality and enlarge labels so they are easily readable.**

**Author response:** Thank you for highlighting this.

**Author action:** All diagrams have been updated with higher resolution and enlarged font sizes for readability.

**Reviewer#2, Concern # 3: There are small grammatical issues and inconsistent tenses (e.g., “in life-or-death scenarios like autonomous driving, but in safety-critical scenarios…”). A careful language edit would improve clarity.**

**Author response:** Thank you for this helpful observation.

Author action: The manuscript has been carefully edited for grammar, tense consistency, and clarity.

**Reviewer#1, Concern #4: The comparison with recent models (Figure 6) is strong, but adding a short explanation of why CViT outperforms others (e.g., adaptive attention vs. fixed attention) would provide better insight.**

**Author response:** Thank you for the detailed suggestions. We acknowledge the need for clarity.

**Author action:** A short explanation highlighting CViT’s adaptive attention mechanism versus fixed attention has been added with the explanation of Figure 6.

**Reviewer#1, Concern # 5: Some entries are missing DOIs (e.g., refs. [31], [33], [35]). Please revise to follow PLOS ONE reference formatting guidelines.**

**Author response:** Thank you for pointing this out.

**Author action:** All missing DOIs have been added, and references reformatted according to PLOS ONE guidelines.

**Reviewer#1, Concern # 6: . Kindly read and cite these references:**

**1. Sun, G., Song, L., Yu, H., Chang, V., Du, X.,... Guizani, M. (2019). V2V Routing in a VANET Based on the Autoregressive Integrated Moving Average Model. IEEE Transactions on Vehicular Technology, 68(1), 908-922. doi: 10.1109/TVT.2018.2884525**

**2. Sun, G., Zhang, Y., Liao, D., Yu, H., Du, X.,... Guizani, M. (2018). Bus-Trajectory-Based Street-Centric Routing for Message Delivery in Urban Vehicular Ad Hoc Networks. IEEE Transactions on Vehicular Technology, 67(8), 7550-7563. doi: 10.1109/TVT.2018.2828651**

**3. Li, Z., Hu, J., Leng, B., Xiong, L., & Fu, Z. (2024). An Integrated of Decision Making and Motion Planning Framework for Enhanced Oscillation-Free Capability. IEEE Transactions on Intelligent Transportation Systems, 25(6), 5718-5732. doi: 10.1109/TITS.2023.3332655**

**4. Shen, Z., He, Y., Du, X., Yu, J., Wang, H.,... Wang, Y. (2024). YCANet: Target Detection for Complex Traffic Scenes Based on Camera-LiDAR Fusion. IEEE Sensors Journal, 24(6), 8379-8389. doi: 10.1109/JSEN.2024.3357826**

**5. Wang, J., Wang, H., Song, J., Chen, X., Guo, J., Li, K.,... Huang, B. (2025). Knowledge-guided self-learning control strategy for mixed vehicle platoons with delays. Nature Communications, 16(1), 7705. doi: 10.1038/s41467-025-62597-x**

**6. Lu, Y., Chen, S., Zhang, X., Pan, X., Gang, Y.,... Wang, C. (2025). A quantum-enhanced heuristic algorithm for optimizing aircraft landing problems in low-altitude intelligent transportation systems. Scientific Reports, 15(1), 21606. doi: 10.1038/s41598-025-05261-0**

**7. Song, D., Zhao, J., Zhu, B., Han, J., & Jia, S. (2024). Subjective Driving Risk Prediction Based on Spatiotemporal Distribution Features of Human Driver’s Cognitive Risk. IEEE Transactions on Intelligent Transportation Systems, 25(11), 16687-16703. doi: 10.1109/TITS.2024.3409874**

**8. Zuo, C., Zhang, X., Zhao, G., & Yan, L. (2025). PCR: A Parallel Convolution Residual Network for Traffic Flow Prediction. IEEE Transactions on Emerging Topics in Computational Intelligence, 9(4), 3072-3083. doi: 10.1109/TETCI.2025.3525656**

**9. Sun, T., Guo, R., Chen, G., Wang, H., Li, E.,... Zhang, W. (2025). RID-LIO: robust and accurate intensity-assisted LiDAR-based SLAM for degenerated environments. Measurement Science and Technology, 36(3), 36313. doi: 10.1088/1361-6501/adb769**

**10. Zhou, X., Zhao, Z., Shen, J., Liu, Z., Liu, Y.,... Xue, B. (2025). Sparse Aperture ISAR Autofocusing and Imaging Algorithm Based on Log-Sum Regularization. IEEE Transactions on Geoscience and Remote Sensing, 63. doi: 10.1109/TGRS.2025.3560140**

**11. Zhu, B., Tang, R., Zhao, J., Zhang, P., Li, W., Cao, X.,... Li, S. (2025). Critical scenarios adversarial generation method for intelligent vehicles testing based on hierarchical reinforcement architecture. Accident Analysis & Prevention, 215, 108013. doi:** [**https://doi.org/10.1016/j.aap.2025.108013**](https://doi.org/10.1016/j.aap.2025.108013)

**Author response:** Thank you for the suggestions.

**Author action:** The most relevant suggested references ([39-43]) have been cited at appropriate points in the manuscript.

**Reviewer#2, Concern # 1: Please ensure that “Conditional Visual Transformer (CViT)” is written in full only at its first occurrence, and that all subsequent mentions use the abbreviation “CViT” consistently throughout the manuscript (including figures, tables, and supplementary material).**

**Author response:** Thank you for the valuable comment.

**Author action:** CViT has been written in full only at the first occurrence and abbreviated consistently throughout the manuscript.

**Reviewer#2, Concern # 2: Ensure that “Convolutional Neural Networks (CNNs)” and “Vision Transformers (ViTs)” are expanded only at their first occurrence, and that all subsequent mentions use the abbreviations “CNNs” and “ViTs” consistently throughout the manuscript.**

**Author response:** Thank you for highlighting this.

**Author action:** CNNs and ViTs are expanded only at their first occurrence; abbreviations are used consistently throughout.

**Reviewer#2, Concern # 3: In the Conclusion section, please add the proposed results explicitly, with separate values for accuracy, precision, recall, and F1-score for easier understanding by readers. Moreover, kindly include 2–3 sentences highlighting possible future research directions based on your work.**

**Author response:** Thank you for this helpful observation.

Author action: Accuracy, precision, recall, and F1-score are now explicitly presented in the Conclusion, along with future research directions.

**Reviewer#2, Concern #4: In the Experimental Setup and Results section, add the total number of parameters used in this study to provide a clearer picture of the model’s complexity and computational requirements.**

**Author response:** Thank you for the detailed suggestions. We acknowledge the need for clarity.

**Author action:** The total parameter count of the model has been added in the Experimental Setup and Results section.

**Reviewer#2, Concern # 5: Table 6 is presented in the manuscript, but its caption and detailed information are not adequately discussed in the text. Please revise the manuscript to reference and explain the content of Table 6 clearly within the main body of the paper.**

**Author response:** Thank you for pointing this out.

**Author action:** The text has been revised to clearly reference and explain the content of Table 6.

**Reviewer#2, Concern # 6: Please carefully review the numbering of all equations and their citations throughout the manuscript. Some inconsistencies were noted in equation numbering and flow; these should be corrected for accuracy and clarity.**

**Author response:** Thank you for the observation.

**Author action:** Equation numbering and citations have been carefully reviewed and corrected for accuracy and flow.
